# Early Intervention in Psychosis: Effectiveness and Implementation of a Combined Exercise and Health Behavior Intervention Within Routine Care

**DOI:** 10.3389/fendo.2020.577691

**Published:** 2020-10-26

**Authors:** Jo Smith, Lisa A. Griffiths, Marie Band, Rachael Hird-Smith, Briony Williams, Justine Bold, Eleanor Bradley, Richard Dilworth, Dominic Horne

**Affiliations:** ^1^ School of Allied Health and Community, University of Worcester, Worcester, United Kingdom; ^2^ Department of Nutrition, Food and Exercise Science, Florida State University, Tallahassee, FL, United States; ^3^ Moorfields Eye Hospital NHS Foundation Trust, London, United Kingdom; ^4^ Centre for Medical Education, Medical School, Cardiff University, Cardiff, United Kingdom

**Keywords:** early psychosis, health risk behaviors, exercise, cardiometabolic risk, combined exercise and dietary intervention, implementation research

## Abstract

**Aim:**

Young people with psychosis have higher rates of obesity, premature cardiovascular disease, and death compared to non-psychotic peers in the general population due to changes in metabolic regulation linked to antipsychotic medication and adverse health risk behaviors. The aim of this paper is to outline the development, implementation, and evaluation of a combined 12-week exercise and health behavior intervention delivered as part of an Early Intervention in Psychosis (EIP) routine service, within the UK.

**Methods:**

Participants (n = 27) completed a 12-week combined intervention program, engaging in weekly, 90-min sessions comprising a healthy behavior education session (45 min), followed by a facilitated exercise session (45 min). Anthropometric data from participants (n = 26) were collected at baseline, 12 weeks, and 12 months post-intervention. Health behaviors and clinical measurements were assessed at baseline and 12 months.

**Results:**

Mean baseline data suggests participants were at an increased health risk on entry to the program, with elevated values in mean body mass index (BMI; 70% overweight/obese), waist circumference, resting heart rate, and triglycerides. Fifty percent reported smoking daily, 64% ate < 5 fruits/vegetables per day, and 52% of participants were prescribed highly obesogenic antipsychotic medications (i.e., Olanzapine). At 12 weeks and 12 months, no changes were observed in mean BMI, waist circumference or any other clinical variable (p > 0.05). At 12 months, participants reported a positive impact on health behaviors including improved diet, increased physical activity levels, and cessation of substance use (n = 2), alcohol use (n = 2), and smoking (n = 4). Focus groups captured participant experiences, engagement with and satisfaction with the program, including challenges/barriers to program adherence.

**Conclusions:**

The 12-week exercise and health behaviors program supported participants to attenuate their physical health risk which was sustained at 12-month follow-up. Self-reported positive health behavior changes are likely to have contributed to the prevention of excessive weight gain in this high-risk period. The evaluation was designed to have validity for a “real world EIP setting” and reflect the complexity of delivery to this participant group. Evaluation findings influenced subsequent commissioning of the physical health intervention as an ongoing element of routine EIP care within the participant site.

## Introduction

Individuals with serious mental illness (SMI) have reduced life expectancy compared to non-psychotic peers primarily due to cardiometabolic disorders (1). Multiple etiological factors increase the risk of premature mortality in SMI including metabolic disturbance from second-generation antipsychotic medications (2, 3), adverse health behaviors (4, 5), and alterations in cardiometabolic, immune, and hepatic-pituitary-adrenal systems (6). Young people, with limited previous exposure to antipsychotic agents, appear to be particularly susceptible to rapid and pronounced weight gain when antipsychotic medication is initiated. Approximately, two-thirds of individuals with first episode psychosis (FEP) will experience clinically significant weight gain (by ≥ 7%) during the first 12 months of treatment (7). Propensity to cause weight gain differs between antipsychotics but none are weight neutral (2). Olanzapine is associated with the largest weight effects and higher doses are associated with greater cardiometabolic abnormalities (8). Weight gain is a common unwanted side effect of antipsychotic medication but can also be associated with other adverse lifestyle factors, including sedentary lifestyle, poor diet and unhealthy food habits, as well as an underlying genetic susceptibility to weight gain (9). Antipsychotic medications are also known to impair glucose metabolism, increase cholesterol and triglyceride levels and cause arterial hypertension, leading to metabolic syndrome and a higher risk of diabetes and cardiovascular disease (CVD) (10). Consequently, there has been a growing interest in interventions to control or attenuate weight gain and increased risk of metabolic syndrome to prevent diabetes and CVD in FEP.

Specific targets have been identified for prevention, screening, and treatment of diabetes and CVD in SMI, including programs to address obesity, smoking, hypertension, hyperlipidemia, and sedentary behavior (5, 11–14). Clinical Guidelines (CG155,178,185) and a Quality Standard (QS80) from the National Institute for Health and Care Excellence (15) recommend systematic physical health screening and monitoring for CVD risk, particularly for individuals prescribed antipsychotics, and recommend the use of combined health behavior interventions focused on healthy eating, physical activity (PA), and smoking cessation.

Research has shown a positive impact of physical health screening and intervention for participants with SMI and FEP on health risk behaviors, weight management, and physical health outcomes, findings which should decrease risk of CVD (16–25). Other intervention studies found no positive impact on weight and other long-term cardiovascular risk factors (13, 26, 27) including a large-scale study with schizophrenia and schizoaffective disorder which included FEP patients (28). Despite a growing body of evidence regarding the efficacy of physical health screening and interventions, Deenik et al. (29) suggested it is not sufficient to define policy aims targeting physical health, relying on the results of subsequent efficacy studies. Implementation research is needed to help practitioners develop, implement, and deliver effective real-world interventions in routine clinical practice which considers social determinants of health (funding, transport, social support networks) which may inhibit access to quality health care services (30).

Efforts to improve physical health in SMI patients have encountered patient and service-level barriers to implementation of routine physical healthcare screening, monitoring, and intervention. Patient-level barriers are both practical (money to buy clothing/equipment, transport challenges) and psychological, including psychotic symptoms, anxiety, low motivation, and self-efficacy (31, 32). Service-level barriers include lack of resources for equipment purchase, access to appropriate facilities, staff capacity, leadership engagement, organizational change, and financial policy strategies to support adoption and scaling up of successful intervention programs (33, 34). Gaughran et al. (26) concluded: “The search for effective, pragmatic physical health interventions deliverable in health care services remains”. A pragmatic approach, which considers barriers, social determinants of health and enablers of initial and ongoing participation, assumes particular importance when developing interventions for young people with psychosis (30–32). Further research is needed on effective strategies for program design and guidance for mental healthcare teams to support implementation and delivery of routine health behavior modification programs (35–37). This study comprised two key aims: (a) to document the context and factors influencing design, implementation, and delivery of the intervention, and (b) to evaluate the effectiveness of the intervention in improving clinical health markers and reducing adverse health behaviors that increase risk for cardiometabolic disease and premature mortality in an FEP population.

## Materials and Methods

A team of healthcare professionals, researchers and service users were involved in design, implementation, and delivery of the “Supporting Health And Promoting Exercise” (SHAPE) program. Charitable funding from The Health Foundation funded intervention development, implementation, and evaluation. Evaluation employed an effectiveness-implementation hybrid design (35, 38) to assess the implementation of the SHAPE program in routine EIP clinical practice.

### Intervention Design and Implementation

The program team, supported by an EIP service user reference group, met regularly to design the intervention and delivery strategy. Discussions were an iterative process to agree educational and exercise program content, location and mode of delivery, assessment of clinical data and health risk behaviors, staff and peer worker roles, branding/logo for the program, and recruitment of participants. It was agreed that an evidence-based approach would be to customize a successful FEP intervention model established within routine EIP care called the “Keeping the Body in Mind” (KBIM) intervention, developed and evaluated by the Bondi EIP service in Sydney, Australia (21, 22). Utilizing this as a framework, the team customized the program to address key factors and social determinants of health associated with implementation in UK EIP service setting.

Key program factors included the need for the intervention environment to foster a positive social identity and social identification (39). Social identity, defined as a person’s sense of who they are within a group or group membership, has been shown to be a key mechanism underpinning the effectiveness of group-based exercise and weight management programs (39). Social identification, a person identifying themselves as a group member, has been found to lead to greater subsequent effort in a group task (40). Participants were provided with a program t-shirt, drinks bottle, pedometer, and tape measure embossed with the SHAPE logo to foster social identity and identification with the group. The program was delivered at a university-community fitness center, instead of a clinical setting, to provide social engagement with other young people in an age-appropriate environment while still providing a clinical and research infra-structure (41).

Behavior modification, social support, and behavior shaping have all been shown to improve intervention effectiveness in SMI, so were embedded within the program design (11, 42). Social support has been shown to be a key component maximizing weight management program retention (42); therefore, we employed university students to support participants through group exercise sessions and to engage with participants in the fitness center outside of the formal SHAPE program on request. Two EIP peer support workers were recruited to support participant engagement and address and allay concerns about attendance and expectations. Peer support was also encouraged through group exercise sessions and team activities. To extend social support outside the program (42), a carers’ evening was incorporated into the program schedule to provide information about the SHAPE program, an opportunity to view the facilities, and an education session targeting family well-being and healthy eating followed by an optional 3-km walk using pedometers.

Access to transport and program accessibility, as well as suitable attire and footwear, were identified as potential barriers to participation (32). The program was delivered at a city center location easily accessible to buses and trains, and/or parking. Participants were encouraged to make their own travel arrangements; however, transport was provided by EIP staff or a community driver when required. Individuals unable to afford suitable exercise attire were supported financially by local charitable grant funding or personal welfare budget funding to reduce potential self-stigma and group marginalization. Mental health nurses from EIP oversaw program delivery and monitored mental and physical well-being and social functioning and fed back observations at weekly EIP team meetings. This close working afforded EIP teams the opportunity to identify and address individual barriers to attendance.

Maintenance of health behaviors and long-term sustainability were identified as key indicators of success. Components to effectively transition participants toward autonomous decision making about their physical health, including goal setting, practical strategies for food shopping, improving PA self-efficacy, and access to local health and fitness services were embedded within the program. Exercise program design was grounded using aspects of self-determination theory (43, 44), including key strategies structured to foster PA self-efficacy and improve perceived competence by offering a range of mastery experiences with education about monitoring exercise intensity and safe exercise progression (45). It was important to develop independent engagement with exercise during the program. To facilitate this, we offered an informal SHAPE session which allowed members a dedicated time to meet socially and exercise together. Toward the end of the program, care coordinators explored local provision to support transition to local fitness facilities for continuation of participants’ exercise regimens at program termination. EIP care planning included a review of program outcomes and how these could be maintained through continued SHAPE program attendance or using alternative local exercise options. SHAPE participants were offered a free gym membership for 12 months at the university fitness center.

### Combined Exercise and Health Behavior Intervention

The SHAPE program was delivered as one component of the routine EIP care package. The program was a combined exercise and health behavior intervention delivered over a 12-week period in a weekly supervised group setting. Educational and supervised exercise sessions were designed and delivered to increase the participants’ understanding of how health behaviors impact short- and long-term physical health. Educational sessions included topics on: healthy eating (21, 37), substance avoidance (drugs and alcohol plus smoking cessation) (37, 46–48), mindfulness (49) and personal care (sleep management) (50, 51), sexual health (52), dental hygiene (53), and goal-setting (54). A trained nutritionist delivered three education sessions focused on healthy eating (5 fruit/vegetables per day, a balanced diet, portion sizes), menu planning, shopping lists, eating out and healthy snacking designed to enable participants to make healthier food and drink choices to help off-set weight gains. Specialist nutrition experts leading these sessions were shown to have the greatest impact on weight management and cardiometabolic risk reduction in people with SMI (55). Nutrition sessions were designed to be interactive and were supported with leaflets, a snack tasting session, and a free healthy eating cookbook. Participants were offered one-to-one nutrition support sessions following SHAPE program completion.

Following educational sessions, participants engaged in a 45-min exercise session led by qualified fitness experts and supported by university students. Exercise education included: how to use fitness equipment, how to self-monitor exercise intensity and progression, and appropriate exercise training techniques (56). Activities included: circuit training, cardiovascular exercise, weight training, Tai Chi, yoga, Pilates, power walking, and small team games (badminton, basketball and handball). Exercise sessions were designed to: a) educate participants on how to exercise safely and effectively; and b) improve PA self-efficacy and autonomous motivation by using a variety of exercises in different settings (fitness equipment, group training, small team games) (57). Utilizing a broad range of mastery experiences (opportunities to successfully achieve a goal), vicarious experiences (witnessing someone who is similar performing a task), and positive verbal persuasion (providing feedback on task performance) during exercise may improve self-efficacy and influence sustained behavior change (45, 58). Perceived variation of exercises with a range of tasks in different social contexts may improve individual’s felt experience and perception leading to increased autonomous motivation (59, 60). **Table 1** highlights the SHAPE 12-week program schedule.

**Table 1:** SHAPE 12-week education and exercise session program schedule.

Session	Health Behavior Topic	Exercise Session Content	Supporting Tools
1	Introduction to SHAPE	Orientation to Fitness Center	Baseline assessment Tape measure SHAPE T-shirt
2	Goal setting	Cardiovascular training session	Goal setting Monitoring exercise intensity
3	Healthy Eating: Healthy Diet and Portion Sizes	Resistance training session	Cookbook Water bottle
4	Healthy Minds: Anxiety and Depression	Small team games: Badminton/basketball/handball	
5	Healthy Lifestyle: Smoking Cessation	Circuit training	
6	Healthy Lifestyle: Drugs and Psychosis	Individualized training	
7	Healthy Eating: Carer Session Healthy Snack Tasting Session	Walk for Health	Carer’s session—social support Pedometer Cookbook Healthy snack sampling
8	Healthy Minds: Mindfulness	Yoga or Pilates	
9	Small Team Games: Badminton, Basketball, handball		
10	Healthy Eating: Menu Planning, Store Cupboard Items, and Shopping Lists	Tai Chi	Shopping Lists
11	Healthy Body: Dental and Sexual Health	Designing your own workout program Open exercise sessions	Individualized workout program
12	Goal setting, Post-intervention assessment, and Focus Group	Open exercise sessions	Goal setting

The program was delivered to five separate cohorts over a 12-month period. Delivery to multiple cohorts allowed for “real-time” participant feedback to modify the program. Focus groups were utilized to capture participant experiences, engagement and satisfaction with the program, including challenges/barriers to program adherence.

### Participants

Individuals with FEP, from a countywide EIP service, comprising two teams covering separate geographical catchment areas, were invited to participate in the program. FEP is defined as the first time a person experiences a combination of symptoms known as psychosis. In the UK, EIP services provide support typically for up to the first three years after psychosis onset (61). Clinical diagnosis was determined by a team consultant psychiatrist during routine psychiatric assessment using International Classification of Diseases (ICD)-10 (62) criteria for a psychotic disorder. Participant eligibility was determined by a physical health lead nurse in consultation with case managers and individual service users, in the context of routine individualized care planning. Service users aged under 16 years old, or who were pregnant or lactating, or whose psychotic symptoms were not yet well-controlled on antipsychotic medication were unable to join the program. Service users who were unable to join, declined to attend, failed to engage, or dropped out from the program were given the opportunity to attend a later SHAPE cohort when their mental health had stabilized or other factors inhibiting attendance and participation had been resolved.

Participants provided voluntarily written consent for their routine clinical data to be used in the study. This study conformed to the principles of the Helsinski declaration and was granted ethical approval by the University of Worcester Research Ethics Committee (REC approval number: UWEC2014JS1).

### Procedures

#### Assessment of Cardiometabolic Risk and Health Risk Behaviors

On acceptance onto EIP caseload, participants had a routine physical health assessment conducted by a mental health nurse. Clinical measures included: BMI, waist circumference, resting heart rate and blood pressure, glycated hemoglobin (HbA1c), fasting plasma glucose (FPG), blood lipids, and prolactin. Blood lipids included measures of total cholesterol (TC), low-density lipoprotein (LDL-C), high-density lipoprotein (HDL-C), and triglycerides. Abdominal adiposity was assessed using waist circumference where measurements were made at the approximate midpoint between the lower margin of the last palpable rib and the top of the iliac crest (63). Blood tests to determine HbA1c and/or FPG, blood lipids, and prolactin were requested by the patients’ general practitioners and conducted by NHS pathology departments. All blood data were collected from electronic patient records. Physical health markers were assessed against NICE (15) criteria to determine risk levels for cardiovascular and metabolic disorders. All clinical measures were assessed at baseline and 12 months post-intervention as part of routine clinical care monitoring.

Health risk behaviors were assessed using self-reported measures for diet (eating > 5 fruit/vegetables per day; equivalent to 400 g a day based on 80 g portions) (64), tobacco use (current smoker or within last 6 months), alcohol use (Alcohol Use Disorders Identification Test) (65), substance use (yes/no response), and PA levels (Exercise Vital Sign (EVS) (66). Sedentary behaviors were determined as engaging in PA less than < 90 min of moderate PA per week (56). Anthropometric data were collected at week 1, immediately post-intervention (week 12), and 12 months post-intervention.

#### Program Evaluation

Attendance monitoring and semi-structured focus group discussions generated qualitative feedback as part of an iterative process to develop and refine the SHAPE program to meet user needs. Program adherence was monitored by recording attendance and individuals’ reasons for non-attendance and disengagement. Barriers to attendance were recorded and problem solved in SHAPE and EIP team meetings to support program uptake and adherence.

Focus group discussions were conducted after the last exercise session of the 12-week SHAPE program. The purpose of the focus groups was to provide participant observations and feedback on program participation to support evaluation and validate findings through use of participant quotes. Participants were invited, but not required to attend, and were assured it would not affect or influence program participation, continued use of the fitness facilities, or their EIP care or treatment. Focus groups were semi structured using open ended questions exploring participant reasons for joining SHAPE, experiences of the program, barriers and facilitators affecting participation, impact of the program on weight, PA levels, health behaviors and general functioning as well as suggestions for program change/improvement. Focus groups were conducted by the program lead, a clinical psychologist, who was not involved in the running of the SHAPE program but may have been known to some participants from involvement in their care within the EIP service. A facilitator, known to participants, managed recording equipment and their presence was designed to reduce participant anxiety by providing a familiar face. Focus groups lasted between 45 and 60 min and were audiotaped and transcribed verbatim to support coding and classifying to identify main themes and sub themes (67).

#### Data and Program Analysis

Baseline analyses were conducted for the total population and by gender. Repeated-measures analysis of variance was performed to determine differences over time for anthropometric data. Paired sample t-tests were used to determine significant differences from baseline to 12 months post-intervention for clinical markers. Data were analyzed using IBM SPSS Statistics for Windows, Version 25.0 (Armonk, NY: IBM Corp). Results are reported as the mean ± standard deviation (SD) and/or as a percent of the population sample. Probability values < 0.05 were considered significant.

Transcribed text from the focus group data were deductively analyzed drawing on pre-determined program factors including program location and environment, program design, program content, social support and impact (68). Transcripts were reviewed for content and coded for correspondence with key program factors and any new themes that emerged inductively from the data.

Key measures for program cost evaluation included: 1) percent change in body mass (69) and subsequent improvement in quality-adjusted life years (QALY), where the cost of Quality of Life Years has been shown to reduce if patients can (a) avoid 7% weight gain and (b) stop smoking (15); and 2) percent change in prevalence (based on BMI change) of CHD and Type 2 diabetes (70).

## Results

### Participant Recruitment and Program Adherence

**Figure 1:**
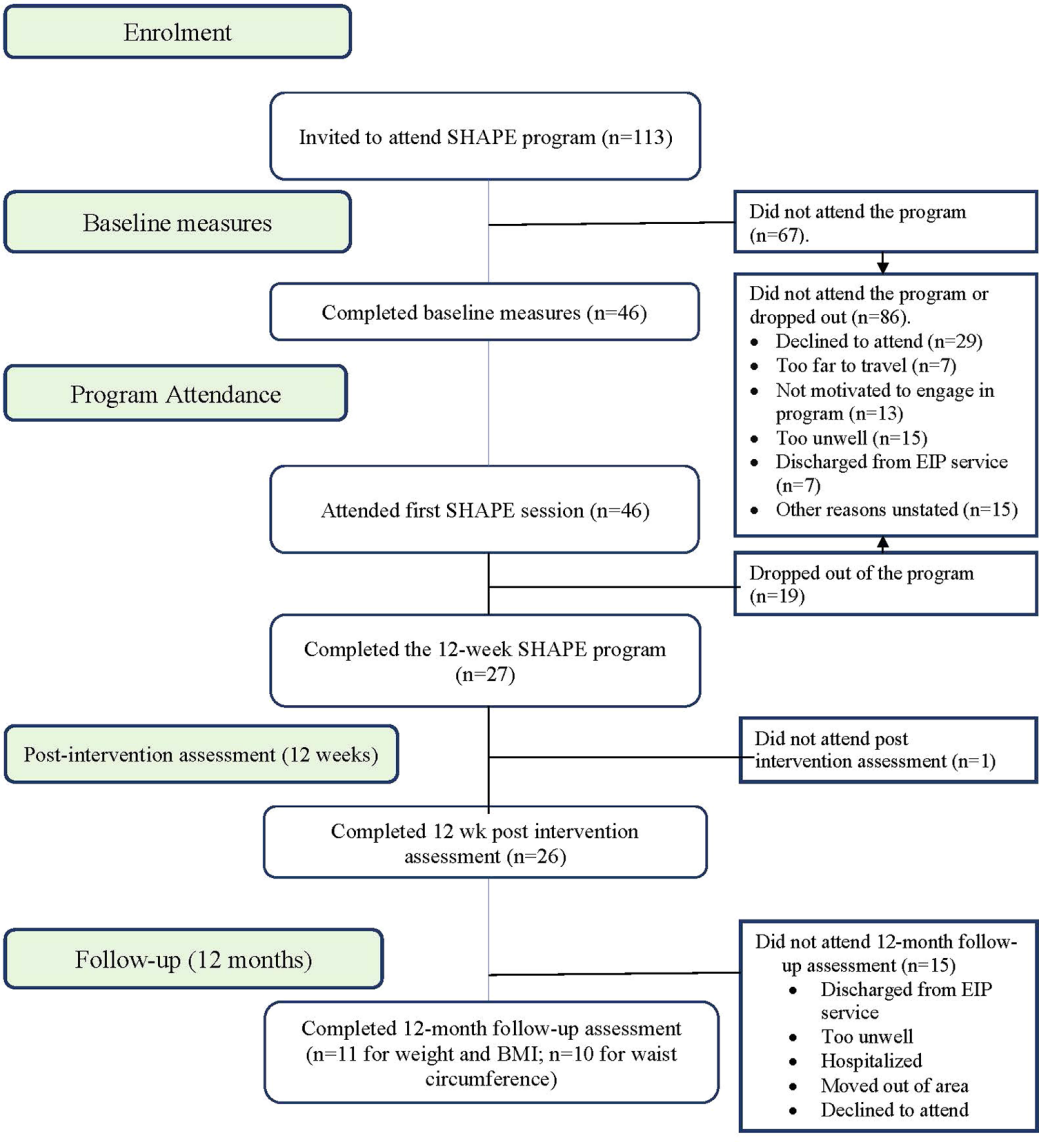
SHAPE Program CONSORT Diagram.

**Figure 2:**
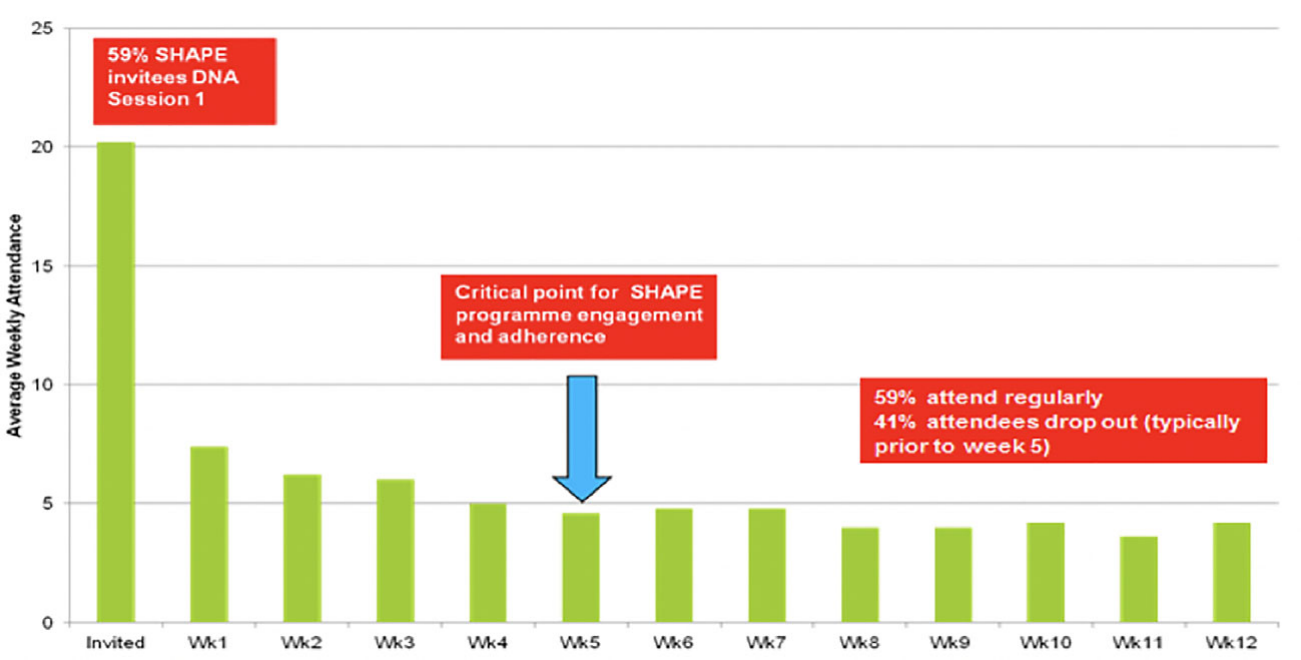
Mean weekly SHAPE program attendance.

**Figure 1** provides program enrollment, program attendance, and post-intervention attendance figures. **Figure 2** shows 59% (n = 67) of invitees did not attend the program. Forty-one percent (n = 46) attended the first SHAPE program session. Week 5 was a critical point for program adherence, where participants attending up to this point were more likely to complete the whole program.

### Participant Characteristics and Clinical Outcomes

Twenty-seven participants aged 18–37 years completed the SHAPE program. Only 26 participants completed the 12-week post-intervention assessment. Completers were those participants who attended a minimum of five sessions. **Table 2** provides demographics, diagnosis, and treatment characteristics by sex for these 26 participants.

**Table 2:** Demographics, diagnosis, and treatment characteristics by sex.

Characteristic	Total (n = 26)	Males (n = 18)	Females (n = 8)
Age (y) M(SD)	27.7 (5.1)	24.0 (3.9)	25.7 (5.4)
**Duration since onset**			
0–6 months	10 (38.5%)	6 (33.3%)	4 (50.0%)
>6 months	16 (61.5%)	12 (66.7%)	4 (50.0%)
**Ethnicity (%)**			
White British	22 (84.6%)	16 (88.9%)	6 (75.0%)
Asian	2 (7.6%)	2 (11.2%)	0
Chinese	1 (3.8%)	0	1 (12.5%)
Undeclared	1 (3.8%)	0	1 (12.5%)
**Primary SCID diagnosis**			
FEP	11 (42.3%)	7 (38.9%)	4 (50.0%)
Schizophrenia	11 (42.3%)	9 (50.0%)	2 (25.0%)
Bipolar	2 (7.7%)	1 (5.6%)	1 (12.5%)
ARMS	1 (3.8%)	0	1 (12.5%
Non-organic	1 (3.8%)	1 (5.6%)	0
**Antipsychotic Treatment (%)**			
No medication	1 (3.8%)	1 (5.6%)	0
Aripiprazole	3 (11.5%)	1 (5.6%)	2 (25.0%)
Clozapine	10 (38.5%)	10 (55.6%)	0
Olanzapine	5 (19.2%)	2 (11.1%)	3 (37.5%)
Quetiapine	5 (19.2%)	2 (11.1%)	3 (37.5%)
Risperidone	2 (7.7%)	2 (11.1%)	0
**Antidepressant Treatment (%)**			
Citalopram	3 (11.5%)	2 (11.1%)	1 (12.5%)
Sertraline	2 (7.7%)	1 (5.6%)	1 (12.5%)
Venlafaxine	2 (7.7%)	0	2 (25.0%)
Fluoxetine	3 (11.5%)	1 (5.6%)	2 (25.0%)

**Tables 3** and **4** provide anthropometric and cardiometabolic data at 12-week and at 12-month follow-up. Mean baseline data (n = 26) suggest participants were already at an increased health risk due to elevated values in mean BMI (81% were overweight or obese), waist circumference (73% at increased risk), resting heart rate (64%), and blood pressure (58% pre-hypertensive; 27% hypertensive), and 50% met the criteria for dyslipidemia. Analysis of health behavior data showed that 39% had a sedentary behavior, 73% ate < 5 fruits/vegetables per day (~400 g equivalent), 46% reported smoking daily, 35% consumed alcohol, and 12% engaged in drug use. Fifty-two percent were prescribed the most obesogenic antipsychotic medications (Clozapine and Olanzapine). Over 85% had elevated resting blood pressure (> 120/80 mm Hg).

**Table 3:** Comparison of anthropometric measurements at baseline, 12 weeks, and 12 months [M(SD)].

12-week post-intervention						
Variable	N	Baseline	12 weeks		Baseline to 12-week post
Body mass (kg)	26	94.4 (23.1)	95.1 (23.4)		*t* = 0.811, *p* = 0.43
BMI (kg.m^−2^)	26	30.7 (7.2)	31.0 (7.4)		*t* = 0.967, *p* = 0.34
Waist circumference (cm)	26	98.1 (17.0)	99.2 (16.8)		*t* = 0.757, *p* = 0.46
**12-month post-intervention**						
**Variable**	**N**	**Baseline**	**12 weeks**	**12months**	**Baseline to 12-month post**
Body mass (kg)	11	99.0 (30.8)	100.0 (31.8)	100.8 (31.2)	*F* = 0.551, *p* = 0.50
BMI (kg.m^−2^)	11	33.0 (9.6)	33.3 (9.8)	33.7 (9.9)	*F* = 0.584, *p* = 0.48
Waist circumference (cm)	10	98.2 (21.0)	99.6 (23.7)	99.7 (25.2)	*F* = 0.258, *p* = 0.75

**Table 4:** Cardiometabolic risk markers and lifestyle behaviors at baseline and 12 months post-intervention [M(SD)].

Variable	N	Baseline	12 months	Mean Change	95% CI Lower, Upper	*P value*
Body mass (kg)	11	99.0 (30.8)	100.8 (31.2)	1.86 (7.3)	−3.02, 6.7	0.42
Body mass index (kg.m^−2^)	11	33.0 (9.5)	33.7 (9.9)	0.7 (2.5)	−1.02, 2.3	0.41
Waist circumference	10	98.2 (21.0)	99.7 (25.2)	1.5 (8.0)	−4.3, 7.2	0.57
Systolic blood pressure (mm Hg)	11	121.6 (12.9)	124.2 (15.7)	2.5 (17.5)	−9.24, 14.3	0.64
Diastolic blood pressure (mm Hg)	11	80.9 (8.7)	82.6 (6.7)	1.7 (10.6)	−5.4, 8.9	0.60
Resting heart rate (beats.min^−1^)	10	86.8 (23.3)	89.9 (22.2)	3.1 (20.4)	−11.5, 17.7	0.64
Total cholesterol (mmol.L^−1^)	6	4.3 (0.9)	4.1 (0.9)	−0.2 (0.9)	−1.2, 0.7	0.48
HDL cholesterol (mmol.L^−1^)	5	1.0 (0.2)	1.6 (1.5)	0.6 (1.5)	−1.2, 2.4	0.58
Triglycerides (mmol.L^−1^)	2	2.4 (2.2)	2.9 (2.9)	0.4 (1.5)	−5.7, 6.6	0.64
HbA1_C_ (mmol.mol)	6	31.9 (14.6)	37.7 (9.7)	7.0 (11.4)	−5.6, 19.7	0.22
Prolactin (mIU/L^−1^)	4	1083.5 (707.3)	433 (239.1)	−650.5 (595.3)	−1737.8, 436.8	0.15

At 12-week post-intervention (n = 26), there were no statistical changes in mean body mass, BMI, or waist circumference (*p* > 0.05). Seven participants maintained weight (±1 kg). Seven participants decreased weight (1.3–8.4 kg); four of whom lost > 5% of their body weight. Twelve participants increased weight (1.3–9.6 kg). Three participants (11.5%) exceeded the Lester UK guidelines of > 5 kg within 3 months (71), of whom, two (8.0%) exceeded the cut off for clinically meaningful weight gain > 7% of their bodyweight (16). Both participants were prescribed Olanzapine.

At 12-month post-intervention (n = 11), there was no statistical change in mean BMI, waist circumference, or any other clinical variable (*p* > 0.05). Two participants maintained weight (±1 kg). Three participants decreased weight (−2.2 to 6.6 kg); 2 of whom lost > 5% of their body weight. Six participants gained 1.3 to 3.4 kg. One individual (9.0%), taking Olanzapine, exceeded > 7% of their bodyweight and the Lester UK > 5-kg threshold. Multivariate analysis revealed no change over time for body mass *(F* =1.009; *p* = 0.403, ηp^2^ = 0.183), BMI *(F* = 0.792; *p* = 0.482; ηp^2^ = 0.150), or waist circumference *(F*= 0.165; *p* =0.851, ηp^2^ = 0.40).

At 12 months, positive impacts on health risk behaviors included: 5 (45%) participants eating > 5 fruit/vegetables daily (~400 g equivalent), 1 (9%) ceased substance use, 2 (18%) ceased alcohol use, 4 (36%) ceased smoking, and 4 (36%) were less sedentary.

### Participant Feedback

Focus group quotes to illustrate main program factors are presented within **Table 5**. One main theme: “impact on wider functioning”, and two subthemes: “feeling better/sense of accomplishment”, and “program commitment”, were constructed inductively from the data during analysis.

**Table 5:** Participant focus group feedback relating to program factors.

Program Factors	Sub-Themes	Participant Feedback (positive)	Participant Feedback (negative)
**Program location, access, timing and environment**	**Location and Access:** **Environment:** **Timing:**	*“it was fairly easy to get to … less than 5-minute walk from the train station.”* *“relaxed environment … not as busy as some gyms … You’re in control of what you’re doing, and it’s just a good experience.”* *“there’s other people that come at the same time, … there’s other people working out as well, so you feel like you’re in a normal environment.”* *“Because it’s a Uni- it’s got that extra edge to it. All the students walking around, learning, smart.”* *“it’s not too early … you can travel after rush-hour…”*	*“… I have to travel quite a way to get here.”* *“… it costs quite a lot of money to get here, so it may be easier for me to find a local gym.”*
**Program design and content:** **Health Behavior education sessions**	**Design:** **Content:**	*“it was kind of an open discussion on options we have over our lifestyle rather than like you’re not eating this or you’re not eating that or get out and do that! … make your own decisions based on our recommendations … which was good.”* *“Every week was different … It wasn’t the same old* *thing, it was very varied.”* *“I like the talking and nutrition part.”* *“Yes, saying how big the portion is, you only need a* *small amount where I thought you needed a lot. … Yes, the nutrition ones were good.”* *“I liked the tasting session as well, when we tried new things, healthy snacks….”* *“I also liked learning about drugs, I found it interesting.”* *“new ideas from sessions before the gym.”*	*“I much preferred the exercise part. I already knew a lot of what was being said. I would have preferred to spend longer doing the sports things, but I understand that some people found it really beneficial so I didn’t mind.”* *“At one point he suggested dips and crackers, as a substitute for crisps and things. I noticed if you bought a tub of dip or whatever, it would go off within 2 days, and you have to have quite a lot of crackers over the two days, rather than having it once a week or twice a week, you are having to have it quite a lot to use it all and not waste it.”* *“I found mindfulness difficult to do as I was thinking about lots of things.”* *“I have tried the vapour cigarettes but I didn’t like them.”*
**Program Design and Content: Exercise Sessions**	**Design:** **Content:**	*“I learnt how to use the fitness equipment. … should be alright for me to work the machinery on my own.”* *“Learning the new equipment, … getting to grips with that because it’s something that’s alien to me.”* *“I think it’s worthwhile doing it (goal setting), so you know what you’re trying to achieve, rather than just coming in and not really having a goal.”* *“… the gym an adequate length, and we’ve got the opportunity to stay after the session is over, so the length of that isn’t a problem.”* *“using the gym facilities … particularly like the rowing* *machine and treadmill.”* *“Handball. … that was my favourite. That broke up the programme as well, doing something a bit different every time, so that was quite good.”* *“My favourite activity … basketball.”* *“I enjoyed Pilates, it was relaxing. Good for* *strengthening the muscles.”* *“ … body balance has been my favourite”*	*“I was worried about using the* *equipment, and how to use it.”* *“I actually used the gym probably for* *about half an hour longer. It seemed a bit too short.”* *“I found the weights difficult.”* *“Pilates was okay, not really something I would normally do.”* *“I found the Tai Chi difficult to follow.”*
**Supporting Tools:** **(pedometer, water bottle, tape measure, cookery book**		*“The books help … something else to focus on, and to try things that you wouldn’t ordinarily try.”* *“I got given a pedometer … then I knew I supposed to have 10,000 a day so that kind of came quite addictive then.”* *“I still check my step counter all the time”* *“I wouldn’t have walked this far had I not had to do it, and it (step counter) made me increase the activity level, and walking around outside of the times as well.”*	*“it (step-counter) could have been done earlier. … we could have looked to see how much everybody had walked, and do it week on week to see if we’ve increased. Or do it start to end and see the progress.”* *“….an app would be really useful to log activity every day or to say everyone get something like my fitness pal like an app that already exists.”*
**Program Engagement**		*“I was hesitant at first, not sure whether I would like it or not, whether there were people there I wouldn’t get on with, so I was a bit hesitant, but eventually said yes.* *“I remember there were people waiting in the entrance on the first week, telling us where to come and introducing us, so that was quite handy.”* *“I was a lot more unstable to start with, as the programs gone on my mood has become more stable.”* *“I was suffering from really bad anxiety and panic attacks, and I really didn’t want to go. I really had to force myself to go, but I survived the first time, which made me persevere.”* *“After the first week of doing it, I wanted to keep doing it, to keep helping me.”* *“I don’t think there is anything that would put me off from what I experienced in the first week so if someone would drop out its probably to do with their own problems rather than the group.”*	*“sometimes, for someone with psychosis or schizophrenia, sometimes it can be difficult to cope.”* *“ the size of the group at the start was a bit overwhelming.”*
**Program adherence**	**Structure/Routine:** **Feeling better/sense of accomplishment:** **Program commitment:** **Intrinsic Motivation:**	*“I quite liked how it structured my day, … you feel like you’ve accomplished something after coming and that’s good for my self-esteem.”* *“It feels good to have a constant routine coming to the gym every Thursday. I have more energy.”* *“it is good and has got me into a routine again.”* *“also, the structure to your day, when I get home I feel like I’ve done something.”* *“I feel differently when I haven’t been like I feel less productive, it’s part of my routine now.”* *“once I’ve done the exercise, and I’ve done it, I feel loads better, like a weight has been lifted, it enables me to carry on for the day, for the rest of the week. It really motivates me, just by the fact that it lifts me.”“…I do feel better immediately after the exercise.”* *“I always feel like on Thursday is like a really happy time because I finish SHAPE then I feel rewarded for going out and doing something … I feel like I have accomplished something after coming and that’s good for my self-esteem.”* *“Although it got better each time, it wasn’t the nicest, but once I got into it, into the session, it got easier and then gradually over the weeks it got easier still.”* *“the two things that have changed the most is my concentration and mood … I was a lot more unstable to start with, as the programmes gone on my mood has become more stable”* *“Sometimes I wake up and I don’t feel like doing anything, but because I’m committed to coming I force myself to go.”* *“I didn’t feel well, but I thought I should go as it’s the last week, … I felt better afterwards.”* *“quite good having that on Thursday then I find myself going to the gym on Friday and Saturday because it’s motivating.”* *“there’s loads of things. It’s fun, it’s worthwhile doing, you make friends, it gets you fit, helps with your motivation.”* *“I think that my motivation has got better because before I … just sat in my room at home.”*	*“I had to force myself to come, to make myself do it, because I thought once I was here, I would go and do it, but it seemed like such an effort at times to just get in, and be here.”*
**Social identity and group cohesion**		*“it was good to start off with a chat, and see which people were with their t-shirts on.”* *“you could tell who else was in the group as well, and kind of feel together, sense of identity.”*	
**Social support**		“*… I felt like I’d made a friend, and then more friends, and it was easier than just being on your own, because you felt like you were going with somebody.”* *“as the group got smaller, you kind of got to know people better … you had regular contact with a regular person which was nice.”* *“… to see other people in your situation benefitting from the same things as you, it’s quite reassuring.”* *“the group is much better, because you can do more things. …The group was one of the reasons I wanted to do it, because I wasn’t really seeing anyone, so that was the main thing – to do something that I enjoyed but also improve socially I suppose.”* *“… get to know people better, and you find out you might have other activities that you do together so, that helps and you learn things about each other which is quite fun.”* *“… you can’t do sports on your own, so the sports I enjoyed.”* *“the person who I came along with grew in confidence throughout and more importantly gained a friendship has led to the two of them carrying on at the Gym and meeting up on a weekly basis.” (peer support worker)*	*“I’ve been talking to X about membership, so then I would have someone to go with, because I wouldn’t do it on my own.”* *“… I can go by myself. I have been to a gym outside of this, I don’t really mind going on my own.”*
**Carer support and feedback**		*“My husband helped– we changed our diet together.”* *“Yes, they can see how it’s helped me, it’s improved my confidence.”* *“My dad said I’ve lost weight, he usually only says that if it’s true.”* *“… It has just transformed him. Not only is he back in shape – almost to his old self – but he is so much more positive and confident. … He is taking much more interest in his own wellbeing which is what SHAPE is about – not just the exercise. He is looking good so we have been clothes shopping and he taking an interest in how he dresses … The 12 weeks are up but he remains just as motivated which couldn’t be better illustrated by the fact that he, and another fellow on the course, are keeping up the same routine and are still going to the centre. From my point of view, SHAPE has given me my son back.” (carer)*	
**Impact on Clinical Outcomes: weight, BMI, heart rate**		*“I haven’t eaten less, but my waist circumference has gone down, and my BMI has gone down.”* *“I have lost a stone so far, I can also feel it in my clothes.”* *“The sessions have been worthwhile and I have gotten more fit and I have lost weight.”* *“My heart rate is a bit lower.”*	*“well I have actually put on weight but I think I have put on muscle in my legs and my belly.”* *“not everyone wants to just lose weight so like if I said I want to get stronger then maybe you could take arm measurements … instead of just saying you have gained or lost weight.”*
**Impact on health risk** **behavior:** **Diet and healthy eating**		*“I am trying to eat more fruit and veg now.”* *“I have cut down on eating and I am eating healthier food.”* *“we try to get extra portions of vegetables into our meal, and find new, creative ways to incorporate them … and extra fruit and things like that.”* *“because of the nutrition side of things, I’m trying to concentrate more on healthy eating and things.”* *“I did some research, and found that wheat sort of puffs you up, so trying to cut that out.”* *“I’m doing more cooking on my own now. I’m eating much more now but I feel healthier, my appetite has increased.”*	
**Impact on health risk** **behavior:** **Physical activity and Sedentary behavior**		*“I can walk quicker without losing breath. Before I’d have to walk slow.”* *“Feel fitter, easier to move around without getting out of breath.”* *“I can actually run up the stairs now.”* *“it’s good to have exercise as a form of release or, as I know I do feel better if I am having a really bad day, if I exercise, the rush or whatever you get, you feel better for like an hour or two and removes me from it for a while.”*	
**Impact on health risk behavior:** **Smoking**		*“I was a smoker before and even though I really enjoyed smoking did reluctantly want to stop, I have now”* *“I reduced smoking from 30 a day to 5 a day over the last 2 months.”* *“I just feel a bit like I have a bit more energy. I think my heart’s a lot healthier now because like before obviously smoking and not exercising for quite a while, I feel like my stamina is better.”*	
**Impact on Wider Functioning**		*“I am starting swimming, gym and exercise classes. The SHAPE gym helped me get motivated.”* *“now I quite like to cycle down the road … then walk the rest of the way into town.”* *“It has made me change my routine on other days as well. …. I’ve started doing it in the morning, then setting up my day with it, just to get that level of activity and to make me feel just a little bit better.”* *“I had a car accident about 5 years ago, and my knees have been weakened since, and I think walking here and the activities we’ve been doing here has helped strengthen them.”* *“I think that my motivation has got better because before I felt like I was just sat in my room at home.”*	*“I still struggle with my fears and bad feelings. At home I’m always so-so, when people ask me how I am I say so-so. I haven’t noticed a big change.”* *“I was struggling to continue after the weeks weren’t taught, it was a bit more of a push to actually get there, for me anyway.”*

## Discussion

The current study sought to implement and evaluate a combined exercise and health behavior intervention within routine care practice in a UK EIP service. The SHAPE intervention represents the first UK evaluation of a combined exercise and health behavior intervention specifically targeting FEP to develop positive health behaviors and improve weight maintenance. The study showed that young people with psychosis are already at an increased risk for CVD and metabolic disorders at time of diagnosis, and early intervention to address their physical health may improve health behaviors and attenuate weight gain at 12 weeks with sustained benefits at 12-month follow-up. The combined exercise and health behavior program was offered as a core element of routine individual EIP care planning, but program participation was affected by patient choice, low motivation, geographical constraints, and intrusive mental health symptoms including social anxiety, low mood, and persistent psychotic symptoms.

### Changes in Physical Health Outcomes

Participants in this study had elevated anthropometric and clinical markers at baseline indicative of increased CVD risk, likely to be linked to self-reported sedentary behavior, smoking, poor diet, high rates of obesity (4, 5), and antipsychotic medication (7). This aligns with previous research in the USA and Australia which has demonstrated a high prevalence of metabolic syndrome in people with schizophrenia at time of diagnosis and with illness progression (~35%–40%) (72–74); and as a consequence, an increased risk for CVD, impaired daily functioning, and premature mortality (72, 75–77).

As far as we are aware, there are only five small-scale studies internationally (16, 17, 21, 24, 41) which have specifically recruited from EIP services. Our findings concur with similar combined interventions in FEP in Australia, which also demonstrated attenuation of weight gain and lower BMI than controls at 3 months post-intervention (16, 21) as well as self-reported positive health behavior changes in PA, fitness, and diet (21). The SHAPE intervention achieved comparable outcomes and mitigated the expected rise in BMI and associated risks for type 2 diabetes and CHD typically observed in an FEP cohort. SHAPE is the first UK EIP intervention study UK to show that effects are maintained at 12-month follow-up, which, if sustained, would reduce associated longer-term health care and QALY costs. Without intervention, we would expect an FEP group to show additional weight gain over 12-month follow-up based on FEP weight gain projections over time (7).

Two other EIP intervention studies, in Canada (17) and the UK (24), involved an exercise training program only. Abdel-Baki et al. (17) employed a 14-week aerobic interval training (AIT) program and reported a decrease in waist circumference and improvements in cardiorespiratory fitness (*p* < 0.05). By comparison, SHAPE participants did not show a decrease in waist circumference but maintained mean waist circumference at 12-week and 12-month post-intervention. Firth et al. (24) involved a 10-week individualized supervised exercise program but failed to show any impact on body weight, BMI, or waist circumference although they did report improvements in negative symptoms and social functioning post-intervention. The latter study achieved a much higher program retention rate (81%) than other EIP intervention studies, including this study, but found adherence to unsupervised exercise was halved following program cessation. PA levels decreased significantly, and symptomatic improvements were only maintained for those who continued to exercise at 6-month follow-up (25). The remaining EIP intervention study in Denmark has only published a qualitative analysis to date (41). More research is needed to understand the sustained effectiveness of combined health behavior modification and exercise behavior change on weight maintenance and BMI in reducing cardiometabolic risk in FEP (21) and how changes in exercise and health risk behaviors may mediate these effects and can be successfully maintained in the longer term.

The SHAPE intervention appeared to be effective in attenuating antipsychotic induced weight gain for the majority (75%) of participants. Just under 8% of participants at 12 weeks and 9% at 12 months gained more than 7% of their bodyweight. Using the Lester UK guideline criterion (71), 12% participants were above the cut off at 12 weeks and 9% at 12 months. This is considerably lower than that observed in the Álvarez-Jiménez et al. (16) FEP intervention study where 39% of intervention participants had increased their baseline weight by more than 7% at 3 months. The importance of weight maintenance to reduce the risk of all-cause mortality was highlighted by Prospective Studies Collaboration et al. (78), stating that preventing weight gain from 28 to 32 kg.m^−2^ during early middle age would yield about 2 years of additional life expectancy. The cost of QALY has shown to be reduced if patients can avoid a 7% weight gain (15). Weight losses of as little as 5% of body weight in individuals at risk of metabolic syndromes can also result in clinically meaningful reductions in morbidity and risk of early mortality (79). In this study, 4 participants at 12 weeks and 2 participants at 12 months had decreased > 5% body mass. It may be plausible to expect that reductions in body weight for these participants could also result in corresponding reductions in morbidity and premature mortality.

### Changes in Health Risk Behaviors

Baseline self-report data identified a few adverse health risk behaviors among participants, notably: sedentary behavior, poor dietary intake, and smoking. Positive behavior changes in eating, PA, smoking, and substance use were reported by several participants at 12-month follow-up. These changes are important as they are among the top eight risk factors (alcohol use, tobacco use, high blood pressure, high body mass index, high cholesterol, high blood glucose, low fruit and vegetable intake, and physical inactivity) that account for 61% of cardiovascular deaths and combined, account for over three quarters of ischemic heart disease worldwide (80). The same WHO report identified that reducing these eight risk factors would increase life expectancy by almost five years.

Five participants reported positive changes in their eating habits reflected in eating 400 g equivalent of fruit/vegetables daily which is an important outcome in relation to tackling obesity and reducing the risk of CVD, diabetes, and premature mortality. Adequate consumption of fruit and vegetables reduces the risk for CVD, stomach cancer, and colorectal cancer (81, 82). Similarly, increases in PA are important in people with psychosis who have been shown to be more sedentary (83, 84) and, therefore, at greater risk of obesity and cardiometabolic diseases than their non-psychotic peers (85).

Four participants ceased smoking. Cessation of and reductions in smoking are also important as this has been identified as the most important risk factor for poor health and reduced life expectancy. It is the largest contributor to preventable illnesses including cardiovascular and respiratory diseases and explains much of the excess mortality and reduced life expectancy of people with mental health problems (46, 86). The benefits for smoking cessation and reduction are not just in relation to long term health risks but can also contribute to more immediate benefits in terms of lower stress levels, reduced financial burden associated with the costs of smoking, and improvements in mental health (87, 88).

Alcohol use has also been identified as one of the top five global risks for burden of disease where it is jointly responsible for one fifth of all Disability Adjusted Life Years (DALYs) (years of life lost due to premature death plus years of healthy life lost due to illness and disability), and one quarter of all deaths in the world (89). Two participants had ceased alcohol use at 12 months. Reducing use of alcohol would be expected to have a positive impact on life expectancy by nearly 5 years (89).

Focus group participants attributed weight, exercise, and health behavior changes to the SHAPE program and commented on wider beneficial impacts on their mental health including mood, motivation and functioning. Positive self-reported health behavior changes at 12 months are likely to have contributed to weight attenuation and other physical health benefits observed. Limitations of the study design preclude our ability to show definite associations between health behavior changes and changes in weight, BMI, and other cardiometabolic measures observed over time.

### Program Engagement, Drop Out, and Adherence

Program recruitment was time consuming and required clinical team members to prioritize SHAPE involvement alongside other clinical roles. Young people with psychosis can experience many difficulties associated with their mental health including psychotic symptoms, social anxiety, and poor motivation (32). For some, the prospect of a group-delivered program was particularly daunting. Feedback from participants highlighted challenges associated with travel time and cost, group size, social anxiety, mood instability, difficulties coping, and maintaining motivation. Two thirds (59%) of invitees did not attend the first session due to lack of interest, poor motivation, poor mental health or travel concerns. Timing of program sessions was important where participant feedback stated that anything before 11 am was difficult, which led to scheduled sessions being moved to late morning to support attendance. Forty-one percent of participants dropped out of the program. This is not unique to SHAPE and appears to be a common observation in other EIP physical health intervention studies. STEPWISE self-management education program (Holt et al., 2019) also found program recruitment challenging, where 17% of recruits did not attend any sessions and only 23% attended all seven core and booster sessions. Abdel-Baki et al. (17) reported a drop out rate of 36% where only 64% of their participants completed the training program. Curtis et al. (21) reported attrition rates of 62% for the KBIM intervention and 52% for their standard care comparison group. In the COPUS intervention trial (41), 38% discontinued the intervention with the majority dropping out prior to the start or early in the program and only 23% of participants attended the final session of their 8-week program. Disengagement is a common challenge for EIP services and in FEP studies and has been linked to a number of factors including duration of untreated psychosis, symptom severity at baseline, insight, substance abuse, and lack of family involvement (90). Findings from the study identified the need for future program design to be cognizant of social determinants (finance, transport, support networks) influencing access to health care services and sustained program engagement.

Some participants were motivated and travelled considerable distances to attend weekly sessions, although, travel was an issue for several participants. The EIP service serves a rural catchment area (~630 square miles) which is challenging when identifying a suitable program location. Holt et al. (2019) supported program access using taxis which EIP staff found helpful but commented that participants were unlikely to have attended without this provision and doubted the financial sustainability in routine practice (34). When considering program sustainability, a single location may be an impediment to attendance, particularly for poorly motivated individuals; utilizing multiple locations may increase program accessibility.

### Participant Experiences of and Feedback on the Program

Focus group feedback provided an iterative service improvement loop and enhanced program evaluation. Participants were positive about all aspects of the intervention and provided helpful feedback on program environment, location, timing, accessibility, and content and how these positively influenced motivation and program adherence. A couple talked about the value of the SHAPE t-shirts in creating a group identity. Several commented on the program delivery style encouraging self-determination and autonomy in relation to health choices, and confidence and mastery in relation to using gym exercise equipment. Participants commented on the different session components particularly appreciating the variety of educational session content and enjoyment of different group sports and particular items of gym equipment. They specifically talked about the value of goal setting, keeping an activity log, and the cookbook and step counter as helpful supporting tools. One participant suggested that step counters could have been introduced earlier in the program to encourage goal setting, monitor weekly activity levels and progress over the program course. Another suggested that an app, like MyFitness Pal, could assist with recording and monitoring activity. A number of health benefits from the program, beyond increasing PA or losing weight were identified. The provision of interactive nutrition sessions, including healthy food “sampling” sessions, encouraged participants to try new foods and consider accessible changes to their diet. The group-based delivery of the program reduced social isolation and encouraged group cohesion and contact between participants, both within and outside of the formal SHAPE sessions which was clearly valued by participants. The program also led to reported benefits in motivation, confidence, social anxiety, self-esteem, and smoking cessation and a broader impact on functioning, routine, and mood. Several participants talked about how the program had been a springboard enabling them to take control of and sustain health behavior changes. Participants valued family support and feedback on observed changes in weight, confidence and functioning. Focus group interviews also allowed exploration of factors affecting program adherence and barriers to participation, which were both logistical (cost, distance, and transport ease) as well as psychological (anxiety, low motivation, poor concentration, coping, and confidence related to mental health). These findings are consistent with several recent qualitative evaluations of health behavior programs with FEP groups which identified common features that were valued by participants and reported similar benefits from and barriers affecting participation and adherence (31, 32, 34, 41, 91, 92).

### Program Design and Implementation

Deenik et al. (29) highlighted a need for effective physical health intervention studies with SMI to be translated into real-world settings, suggesting that research on program implementation in standard care practice is an essential step to translate effective health behavior interventions into routine mental health care. Unfortunately, there remains a “research to real-world” gap where research innovations are rarely effectively adopted and implemented in real-world health care settings (93), Both studies highlighted barriers to effective implementation including poor alignment between researchers, health care systems, and providers. They argued that moving research “from innovation to application” requires local input, using local options, and developed using a sustainable approach that enables frontline workers to continue implementation and delivery. Unlike many previous physical health interventions which started as a randomized clinical trial or research-based intervention, the SHAPE program was designed, implemented, and delivered in partnership with EIP service users, the EIP service team, and local researchers and health care professionals. All team members worked locally, were familiar with geography and infrastructure challenges of a large rural county catchment area, and aware of opportunities and local resources that could be harnessed to maximize intervention program design and delivery.

The customized intervention offered a coordinated, multi-professional, health and well-being intervention program which was designed to encourage participants to meet peer SHAPE participants and university student “exercise buddies” within a positive, youth friendly, socially inclusive setting. Other UK physical health intervention programs in FEP (24) did not include a group exercise component (42) or peer support opportunities (94), which have been identified as important factors in long term exercise program adherence and maintenance. In contrast to the Australian KBIM study (21), the SHAPE program was located at an accessible city center location within a university-exercise setting, which was deemed to be more normalizing than a clinical healthcare setting. Firth et al. (24) and Larsen et al. (41) similarly elected to locate their interventions in community fitness facilities. The delivery of exercise interventions in a clinical setting may engender health and safety concerns without having qualified instructors supervising exercise as well as equipment purchase and appropriate space concerns that might impinge on program scope and flexibility. Mental health care services may need to explore local resources and identify partners, such as exercise referral schemes or other community-based interventions, willing to collaborate to deliver lifestyle interventions outside the typical clinical health setting (37).

Similar to this study, participants in the COPUS Trial (41) stated that they enjoyed engaging in a “real-world” exercise setting and felt they were treated by exercise instructors as “normal”. Instructors and student interns engaged with clients during exercise providing instruction and motivational support; this also provided opportunities to monitor participant progress and engagement during the session. SHAPE participants could arrange to meet exercise buddies to use fitness facilities and follow their individualized exercise program outside of the formal SHAPE sessions. Involving University students in program delivery enhanced the “normalizing approach”, increased the mental health awareness of student instructors, and was evaluated positively by SHAPE participants, several of whom expressed an interest in becoming peer support workers on the SHAPE program or pursuing higher education study opportunities themselves. A combined exercise and health behavior group-based intervention appears to be most impactful for young people with FEP based on published studies to date (16, 17, 21, 24).

### Program Challenges

Initially, attitudes toward the intervention within EIP service were mixed as several team members questioned whether this should be a treatment priority or an appropriate focus for the team, perceiving it as a primary care responsibility. Training sessions with EIP staff were provided by the program team about the importance of early physical health, their role in promoting good physical health (including comprehensive repeat physical health assessment and monitoring), and the need to increase referrals to the SHAPE program and to other intervention services including smoking cessation programs and sexual health services.

Changes to clinical practice were required to ensure standardized monitoring of physical health including: 1) improved physical health monitoring and intervention through comprehensive physical health checks, 2) monitoring, recording and evaluating physiological changes that increase risk of CVD and diabetes, 3) early identification of individuals at high risk for cardiometabolic disorders and referral for specialist intervention, and 4) increased communication with general practitioners to improve physical health care planning. These observations are not unique, Blythe and White (95) reported that London-based mental health nurses (n = 585) received limited educational support in their role to monitor physical health and poor communication between primary and secondary care services impacted their ability to address physical health needs of SMI patients.

### Program Costs and Cost Effectiveness

Currently, there is a lack of cost effectiveness research examining supervised exercise programs in SMI, apart from two large scale unsuccessful intervention trials which reported no evidence for their cost effectiveness (28, 96). No EIP studies to date have reported on cost effectiveness. However, similar programs for diabetes, heart disease, and depression have shown exercise programs can produce large economic benefits (97).

Total setup and annual delivery costs of delivering a 12-week SHAPE program to 4 cohorts per year at 4 locations simultaneously within the County (total reach = 125–150 patients) were estimated at £40,000 pa (2015 prices) at an approximate cost of £260–£400 per person per annum. Implementation costs included staff training, recruitment of a program lead, SHAPE package and materials, venue costs, speakers for educational sessions and transport costs. This model allowed for sustainable delivery and for SHAPE to be delivered as one element in an integrated NHS mental and physical health care package.

Weight maintenance and reduction has been linked to improved life expectancy of 2–3 years and a reduced QALY cost if patients can avoid a 7% weight gain (15, 78). Equally, changes in eating, PA, smoking, and alcohol use reported by several participants at 12-month follow-up could potentially increase life expectancy by almost 5 years (89). This does assume participant engagement and program retention and for reported outcomes to be maintained long enough to benefit from the health states associated with them. Based on this, the estimated intervention costs and QALYs improvements linked to SHAPE outcomes would suggest that the SHAPE program is likely to be a cost-effective and value for money intervention. More research is needed on cost effectiveness of physical health intervention programs in FEP considering the unique barriers young people face when engaging in an exercise and health behavior intervention (31, 32) and their impact on successful program engagement, adherence, and likely outcomes.

### Program Sustainability and Scale Up

Implementation and sustainability in a real-world setting eludes the majority of intervention studies to date and requires greater integration of an intervention program within the organization in which it is being implemented (coordination of resources, staffing) as well ongoing resourcing to sustain programs in the long term beyond pilot intervention trials (34, 98). Our program was successfully integrated into existing UK National Health Service (NHS) care to provide service users education and support to achieve sustainable health behavior change.

SHAPE data was used to make a case for investment and funding for the program to be scaled up and delivered in multiple sites countywide. SHAPE was formally commissioned and has since been rolled out across the County with SHAPE programs running weekly in six localities across the catchment area. The SHAPE model was included as a good practice case example on the NICE shared learning database (99) and adopted by other EIP teams regionally and nationally. The SHAPE physical health lead nurse role has been extended for the whole NHS Trust and a permanent exercise coach was employed to support the SHAPE program. Training on how, when and what to screen when undertaking routine physical health monitoring and the importance of a consistent, unified approach to collecting physiological measures, awareness of risk indicators, and what kinds of interventions are appropriate in response to risks identified has been rolled out for mental health professionals locally. Online lifestyle and nutrition education materials and a manual and toolkit were made available on a bespoke SHAPE program website.

### Strengths and Limitations

A “real-world”, effectiveness-implementation hybrid approach to program evaluation was designed to enhance external validity and transfer to other “real-world” settings, which has been problematic with previous randomized controlled trials. Unlike other EIP physical health intervention studies which have employed a “waiting-list” control group (41) or a comparison “treatment as usual” FEP sample from the same service (24), the KBIM intervention was also embedded into routine EIP care (21) and similarly chose to draw on a comparison group recruited from another New South Wales EIP service who received “standard care”. Without a matched control group, we cannot definitively attribute changes reported to the SHAPE intervention, particularly when delivered as one element in a multi-component EIP routine care package, although participant focus group feedback and self-reported health behavior and exercise changes were attributed by participants to SHAPE program participation.

The participant sample is relatively small, although it is the largest intervention sample of all published EIP studies to date. Access to health records was limited to those participants who provided informed consent, so findings may not be reflective of all SHAPE participants or all people with early psychosis under the care of the EIP service. Current study findings are based on outcomes entirely from one EIP service in one geographical area and thus, a sample bias might exist. The area from which the sample was drawn is a mixed urban/rural county with varying levels of economic and social deprivation which might impact on engagement, adherence, and needs of participants. Moreover, it is possible that participants who declined assessments (and not included in the data sample) might have more risk factors for poor physical health linked to their circumstances and perhaps be less likely to report similar views to program participant peers. Equally, program participants might be more likely to engage with assessments and report benefits.

The results of the clinical data should be interpreted in the context of several important limitations. Measures employed in the study evaluation were restricted to those measures carried out at baseline and repeated annually as part of routine physical health assessment and review processes. Health risk behaviors were only routinely re-assessed as part of an annual physical health review and not available at 12-week post-intervention. Clinical measures from the two EIP teams were not always directly comparable with one another as there was no standardized approach in requesting clinical screening data at baseline or at 1 year into service. General practitioners made individual decisions regarding which blood tests were performed making it difficult to compare across teams and timepoints. To ensure a consistent, unified approach to collecting physiological measures (i.e., tests performed and timing) requires agreement across EIP teams in conjunction with primary care services. Similarly, there was some attrition in the 12-month follow-up data sample where health records were not accessible to extract 12-month follow-up physical health review outcomes. Data on antipsychotic exposure, adherence to antipsychotic medication, as well as any changes in antipsychotic medication type and dosage during the 12-month follow-up period were not captured and may be potential confounds influencing study findings.

A semi-structured focus group may have constrained participant responses by encouraging conformity and strategic biases and inhibiting participants from talking about issues that were perceived as particularly relevant to them, rather than *a priori* issues chosen by the program team. Some focus group participants may have been reluctant to talk about potentially sensitive topics in relation to their experience of other participants, negative consequences, or personal information in relation to impact on weight or health behavior in a group discussion setting. Participants separately consented to participate in the focus groups which not all participants chose to attend. Themes identified in the focus group transcript may be subject to bias and not necessarily reflect the experiences of all SHAPE participants or dropouts. Focus groups were conducted by the team members known to some participants within the EIP service. This may have led to an investigator bias in the way questions were asked and elaborated and a desirability bias on the part of participants affecting their responses. Transcripts were examined for main themes and may be subject to inherent investigator bias as the authors were involved in program design and evaluation.

### Considerations and Learning for Future Physical Health Intervention Programs

Future intervention program design should consider combining exercise with lifestyle interventions that are grounded in behavior change theory to foster self-efficacy and improve perceived competence of program participants. Effective implementation requires close alignment between researchers, health care systems, and community providers to develop a sustainable approach and ensure continued implementation and delivery in routine healthcare. Intervention programs need to be of sufficient duration to provide practical skills for exercise, healthy eating, building self-efficacy and autonomy as well as an individualized transition strategy to sustain program outcomes. Consideration of the unique barriers, particularly funding concerns, that young people describe when engaging in an exercise and lifestyle intervention may be critical to successful program engagement and adherence.

## Conclusion

At the time of diagnosis, SHAPE participants were already at an increased risk for cardiometabolic disorders due to elevated clinical markers and unhealthy health behaviors. The findings suggest SHAPE supported young people with FEP to attenuate their physical health risk following a 12-week combined exercise and health behavior intervention which was sustained at 12-month follow-up. Positive health behavior changes reported at 12-month follow-up may have mediated the sustained weight maintenance and improved physical health outcomes observed. Physical health interventions are needed early in the treatment process to address the increased risk for cardiometabolic disorders in individuals recently diagnosed with FEP. Using an effectiveness-implementation hybrid model, to design and evaluate a combined exercise and health behavior intervention for FEP patients in a real-world setting, may enhance program effectiveness and subsequent uptake of effective interventions into routine care.

## Data Availability Statement

The raw data supporting the conclusions of this article will be made available by the authors, without undue reservation.

## Ethics Statement

The studies involving human participants were reviewed and approved by University of Worcester Research Ethics Committee (REC approval number: UWEC2014JS1). The patients/participants provided their written informed consent to participate in this study.

## Author Contributions

JS, LG, MB, BW, and EB devised the original protocol. JS, LG, MB, BW, EB, JB, RD, RH-S, and DH refined the protocol. JS was the chief investigator and oversaw the study. MB and RH-S recruited participants. MB and LG were responsible for study procedures. JS, BW, EB, JB, RD, DH, and RH-S provided feedback on the recruitment methods and helped refine the study procedures. MB, JB, RD, RH-S, and BW delivered the intervention. LG did the quantitative data analysis. JS did the qualitative data analysis. JS oversaw the data analysis. The writing team consisted of JS and LG who drafted and edited the report manuscript. DH and EB proofread the final manuscript. JS, LG, MB, BW, EB, JB, RD, RH-S, and DH critically reviewed drafts of the manuscript for important intellectual content. All authors contributed to the article and approved the submitted version.

## Funding

The Health Foundation: Shine Award 2014 and Spreading Improvement Award 2015; Postcode Anywhere, Diglis Basin, Worcester provided small grant funding for participant equipment (pedometers, water bottles, and tape measures).

## Conflict of Interest

The authors declare that the research was conducted in the absence of any commercial or financial relationships that could be construed as a potential conflict of interest.

## Abbreviations

CVD, Cardiovascular Disease; EIP, Early Intervention in Psychosis; FEP, First Episode of Psychosis; KBIM, Keeping the Body in Mind; NHS, National Health Service; NICE, National Institute for Health and Care Excellence; PA, Physical Activity; PHE, Public Health England; SHAPE, Supporting Health and Promoting Exercise; SMI, Serious Mental Illness; QALY, Quality-Adjusted Life Years; UK, United Kingdom; WHO, World Health Organization.
